# High Levels of 17*β*-Estradiol Are Associated with Increased Matrix Metalloproteinase-2 and Metalloproteinase-9 Activity in Tears of Postmenopausal Women with Dry Eye

**DOI:** 10.1155/2016/2415867

**Published:** 2016-01-19

**Authors:** Guanglin Shen, Xiaoping Ma

**Affiliations:** Department of Ophthalmology, Zhongshan Hospital, Fudan University, 180 Fenglin Road, Shanghai 200032, China

## Abstract

*Purpose*. To determine the serum levels of sex steroids and tear matrix metalloproteinases (MMP) 2 and 9 concentrations in postmenopausal women with dry eye.* Methods*. Forty-four postmenopausal women with dry eye and 22 asymptomatic controls were enrolled. Blood was drawn and analyzed for serum levels of sex steroids and lipids. Then, the following tests were performed: tear collection, Ocular Surface Disease Index (OSDI) questionnaire, fluorescein tear film break-up time (TBUT), corneal fluorescein staining, Schirmer test, and conjunctival impression cytology. The conjunctival mRNA expression and tear concentrations of MMP-2 and MMP-9 were measured.* Results*. Serum 17*β*-estradiol levels were significantly higher in the dry eye subjects than in the controls (*P* = 0.03), whereas there were no significant differences in levels of testosterone, dehydroepiandrosterone sulfate (DHEA-S), and progesterone. Tear MMP-2 and MMP-9 concentrations (*P* < 0.001), as well as the MMP-9 mRNA expression in conjunctival samples (*P* = 0.02), were significantly higher in dry eye subjects than in controls. Serum 17*β*-estradiol levels were positively correlated with tear MMP-2 and MMP-9 concentrations and negatively correlated with Schirmer test values.* Conclusions*. High levels of 17*β*-estradiol are associated with increased matrix metalloproteinase-2 and metalloproteinase-9 activity in tears of postmenopausal women with dry eye.

## 1. Introduction

Epidemiological data have shown that dry eye becomes more frequent with age in both sexes and that women are at a higher risk of dry eye than men [1–3]. The higher prevalence of dry eye in women has been partly attributed to hormonal changes that occur with menstruation, pregnancy, lactation, menopause [4–7], and use of medications such as contraceptives and hormone replacement therapy (HRT) [8]. Sex hormones have been suggested to play a key role in maintaining ocular surface homeostasis.

Ocular surface tissues have been found to be specific targets for sex hormones. Androgen, estrogen, and progesterone receptors have been identified in human lacrimal glands [9], meibomian glands [9, 10], and cornea and conjunctiva [11, 12]. Androgens influence the structure and function of the lacrimal and meibomian glands and exert a significant anti-inflammatory effect on the ocular surface [13, 14]. In contrast, despite the large number of studies, the impact of estrogen and progesterone on the ocular surface tissues is still controversial [15, 16]. Dry eye in postmenopausal women is characterized by both high and low serum estrogen levels and conflicting results [8, 17, 18] have been reported concerning the effect of HRT on the signs and symptoms of dry eye in women. Whether dry eye in females is caused by estrogen excess or deficiency, androgen deficiency or estrogen/androgen relative imbalance remains to be determined.

Matrix metalloproteinases (MMPs) are a family of proteolytic enzymes that function to maintain and remodel tissue architecture. In addition to their normal roles in tissue remodeling, MMP-2 and MMP-9 are known to be critical extracellular matrix remodeling enzymes in wound healing and diseases of the ocular surface [19]. There are many factors regulating MMP expression, including sex steroids, cytokines, growth factors, and cellular interactions and transformation. Sex steroids such as estrogen and testosterone have been shown to regulate MMP-2 and MMP-9 expression. Previous studies have shown that estrogen administration increases the expression of MMP-2 and MMP-9 in immortalized human corneal epithelial cells and the lacrimal glands of ovariectomized rabbits or rats [20–22]. It has also been confirmed that MMP activity is upregulated by estrogen in other tissues. For example, estrogen stimulates MMP-2 expression in human granulosa-lutein cells and vascular smooth cells [23] as well as MMP-9 expression in human mesangial cells [24]. Testosterone administration, on the other hand, has been shown to decrease MMP-2 activity in the lacrimal glands of ovariectomized rats [22]. Hence, further studies are required to determine the relationship between sex steroid levels and MMPs in humans.

The aim of this study was to determine the serum sex steroid levels, including 17*β*-estradiol, testosterone, dehydroepiandrosterone sulfate (DHEA-S), and progesterone, in postmenopausal women with dry eye. Furthermore, we investigated the relationship between sex steroid levels and MMP-2 and MMP-9 activity in tears.

## 2. Materials and Methods

### 2.1. Subjects

Between January 2015 and July 2015, dry eye subjects were consecutively recruited from the outpatient clinic and normal control subjects were recruited from a health checkup population at Zhongshan Hospital of Fudan University. This case-control study was approved by the local Ethical Committee and conducted in accordance with the Declaration of Helsinki. Written informed consent was obtained from each participant before starting the study procedures.

Postmenopausal women aged over 50 years were recruited and later categorized into two groups: the dry eye group and the control group. “Postmenopausal” was defined as no menses for at least 1 year. During a preliminary visit, medical history was assessed, and a comprehensive ophthalmic examination was performed on all participants to ensure eligibility. Dry eye was diagnosed if the subject fulfilled at least two of the following criteria: Ocular Surface Disease Index (OSDI) [25] score > 20, fluorescein tear film break-up time (TBUT) ≤ 5 seconds, and corneal fluorescein staining score > 3 according to the National Eye Institute (NEI) grading scale [26]. The age-matched asymptomatic control subjects exhibited normal results on all of the above measures.

Exclusion criteria for both groups included (1) ceased menses due to autoimmune disorders, smoking, or hysterectomy; (2) a history of Sjogren's syndrome (SS), diabetes, or other systemic disorders known to affect the ocular surface; (3) a history of HRT, contact lens use, or ocular surgery; (4) the use of any topical ocular medication or systemic medication known to exacerbate dry eye; and (5) the presence of anterior segment abnormality or active eye disease other than dry eye.

### 2.2. Study Protocol

Blood was drawn from each participant by phlebotomists at the Department of Clinical Laboratory, Zhongshan Hospital. Then, tears were collected from the participants, the OSDI questionnaire was administered to the participants, and a series of dry eye tests were performed in the following order: fluorescein TBUT, corneal fluorescein staining, Schirmer test, and conjunctival impression cytology. There was at least a 5-minute gap between each test. Dry eye tests were performed by the same researcher to maintain consistency. Application of artificial tears or other ocular lubricants was discontinued 3 days before each participant's study visit. The temperature and humidity of the examination room were controlled at a range from 20°C to 24°C and from 40% to 50%, respectively. For each subject, the right eye was used for analysis.

### 2.3. Laboratory Blood Analysis

Blood samples were drawn from all participants at 8:00 a.m. following an overnight fast. Serum levels of sex steroids (17*β*-estradiol, total testosterone, DHEA-S, and progesterone) were measured using a chemiluminescence method. The limit of detection for the steroids was as follows: 17*β*-estradiol 5.0 pg/mL (18.35 pmol/L), testosterone 0.087 nmol/L, DHEA-S 0.003 *μ*mol/L, and progesterone 0.095 nmol/L. A serum lipid profile, including total cholesterol, triglycerides, high-density lipoprotein- (HDL-) cholesterol, and low-density lipoprotein- (LDL-) cholesterol, was also obtained as it may influence sex steroid levels [27].

### 2.4. Tear Sample Collection

Tear samples were collected using disposable 5 *μ*L microcapillary tubes (Microcaps; Drummond Scientific Co., Broomall, PA) without anesthesia. Approximately 5 *μ*L of tear fluids was gathered from the inferior temporal tear meniscus from each eye. Care was taken to ensure that the lid margin, cornea, or conjunctiva was not touched, to avoid as much as possible reflex tears. The tear flow rate was controlled during the process, and only samples with a flow rate of 1–5 *μ*L/min were used for further tests. Tears from both eyes were pooled together and transferred into a 1.5 mL Eppendorf tube and then immediately stored at −80°C until further examination.

### 2.5. Quantification of MMP-2 and MMP-9 in Tear Samples

Total MMP-2 and MMP-9 (pro- and active forms) concentrations in extracted tear samples were each determined using commercially available quantitative sandwich ELISA kits (Quantikine; R&D Systems, Inc., Minneapolis, MN). Sample preparation and analysis were performed according to the manufacturer's instructions. Tear fluid of precise volume from each sample was transferred and diluted 1 : 20. The final results were corrected according to the dilution factor.

### 2.6. Assessments of Dry Eye

Dry eye symptoms were assessed using the OSDI questionnaire, which has previously been validated as a reliable method for measuring the severity of dry eye [25]. The OSDI consists of 12 questions about symptoms experienced within the previous week and yields scores ranging from 0 (least severe) to 100 (most severe).

Fluorescein TBUT was measured by instilling 5 *μ*L of 2% sodium fluorescein solution and calculating the time between the last complete blink and the appearance of the first dry spot in the stained tear film. Three consecutive measurements were conducted, and the average value was taken.

Corneal fluorescein staining was evaluated under cobalt blue illumination following fluorescein instillation. Corneal staining was assessed using the NEI scale, where grades of 0–3 were assigned for five regions of the corneal surface, up to a total of 15 points.

Then, Schirmer test (without anesthesia) was performed with sterile strips inserted at the border of the medial to the lateral third of the lower lid margin with the lids closed. The moistened length was measured after 5 minutes.

### 2.7. Conjunctival Impression Cytology

Conjunctival epithelial cells were collected via impression cytology as previously described [28]. Briefly, after administration of topical anesthetic (0.5% proparacaine hydrochloride; Alcon), two sterile membrane filters (6 × 6 mm, Millipore) were gently placed onto the inferotemporal and superotemporal bulbar conjunctiva. Gentle pressure was applied to the filters for 10 seconds using blunt smooth edged forceps. The membranes were then gently removed from the eye and transferred into an Eppendorf tube containing TRIzol reagent (Invitrogen; Carlsbad, CA). The samples were then stored at −80°C until processing.

### 2.8. Real-Time PCR

Total RNA in conjunctival cell samples was isolated using TRIzol Reagent (Invitrogen) and then reverse-transcribed with Prime-Script RT Master mix (Takara, Otsu, Japan). Gene expression was detected by quantitative real-time PCR using primers for MMP-2, MMP-9, and glyceraldehyde 3-phosphate dehydrogenase (GADPH). The primer sequences used were as follows: MMP-2 (sense: 5′-AGCGAGTGGATGCCGCCTTTAA-3′; antisense: 5′-CATTCCAGGCATCTGCGATGAG-3′); MMP-9 (sense: 5′-GCCACTACTGTGCCTTTGAGTC-3′; antisense: 5′-CCCTCAGAGAATCGCCAGTACT-3′); and GADPH (sense: 5′-GTCTCCTCTGACTTCAACAGCG-3′; antisense: 5′-ACCACCCTGTTGCTGTAGCCAA-3′). Reactions were performed using the Roche LightCycler 480 System (Roche, Indianapolis, IN) in combination with a SYBR Premix Ex Taq Kit (Takara) according to the manufacturer's instructions. The relative gene expression was calculated using the Comparative *C*_T_ Method and standardizing levels to GADPH mRNA.

### 2.9. Data Analysis

Statistical analysis was performed using SPSS version 20.0 (SPSS Inc., Chicago, IL). Descriptive statistics were presented as the mean ± standard deviation (SD). The sample size (at least 15 eyes at each group) was determined to detect 20% difference in sex steroid levels, with *α* = 0.05 and *β* = 0.20. Some values of 17*β*-estradiol levels were below the limit of assay quantitation (5 pg/mL). Thus, we categorized subjects by 17*β*-estradiol levels less than 5 pg/mL and by 17*β*-estradiol levels of 5 pg/mL or greater for analysis. Differences in demographics and measurements between the 2 groups were assessed using Mann-Whitney test for continuous measures and Pearson's chi-squared test for categorical factors. Spearman's rank correlation coefficients between parameters were calculated. *P* < 0.05 was considered statistically significant.

## 3. Results

### 3.1. Characteristics of Subjects

A total of 66 postmenopausal women (66 eyes, mean age 62.4 ± 6.8 years) were enrolled in this study. Among the 66 subjects, 44 subjects were identified as having dry eye based on the diagnostic criteria, and 22 normal control subjects were included for comparison. Baseline demographics and clinical characteristics in patients with dry eye and normal controls are presented in Table 1. No significant differences were noted between the 2 study groups in terms of age, duration of menopause, body mass index (BMI), or lipid profile (total cholesterol, triglycerides, HDL-cholesterol, and LDL-cholesterol). All 66 women had normal weight (BMI range: 18.5–25) and cholesterol levels. The results of OSDI, fluorescein TBUT, corneal fluorescein staining, and Schirmer test differed significantly between the dry eye group and the control group (*P* < 0.001).

### 3.2. Serum Levels of Sex Steroids

The serum levels of sex steroids in the dry eye group and the control group are presented in Table 2. This study showed detectable levels of 17*β*-estradiol (≥5 pg/mL) in 15 dry eye subjects (34% of subjects) and 2 normal controls (9% of subjects). 17*β*-Estradiol levels were significantly higher in the dry eye subjects than in the controls (*χ* ^2^ = 4.79, *P* = 0.03). Levels of testosterone, DHEA-S, and progesterone were higher in the dry eye group, but differences did not reach a level of significance (*P* = 0.08, *P* = 0.57, and *P* = 0.34, resp.).

### 3.3. MMP-2 and MMP-9 Gene Expression

**Figure 1:**
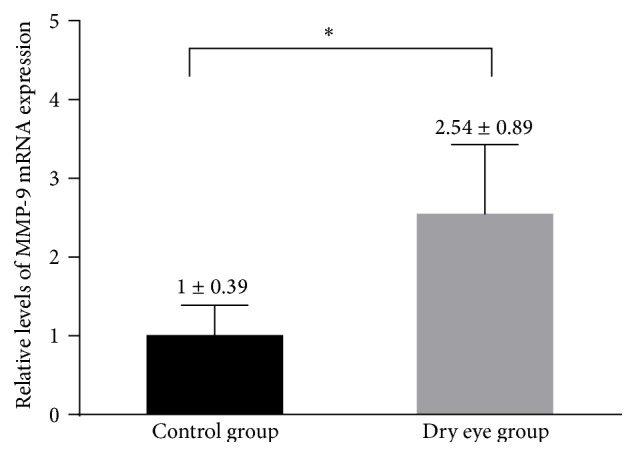
Real-time PCR results of relative levels of MMP-9 mRNA expression in conjunctival cytology samples obtained from patients with dry eye and normal controls. Values were presented as mean ± SD. ^*∗*^Significant difference with *P* < 0.05.

The results of MMP mRNA expression in conjunctival epithelia were obtained by impression cytology from dry eye subjects and normal controls. Significantly higher levels of MMP-9 mRNA were observed in dry eye subjects than in normal controls (*P* = 0.02, Figure 1). MMP-2 transcripts were undetectable in the samples obtained from normal subjects and those obtained from dry eye subjects.

### 3.4. MMP-2 and MMP-9 Concentrations in Tears

**Figure 2:**
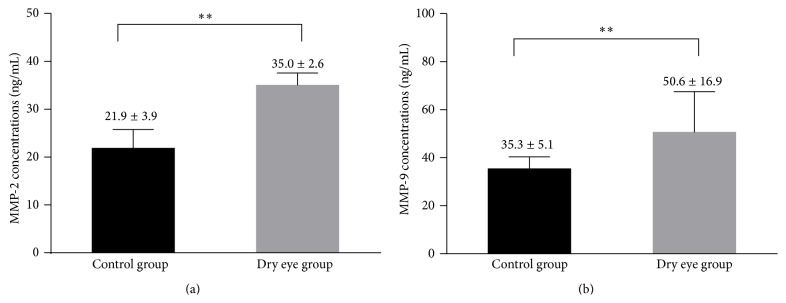
The concentrations of MMP-2 (a) and MMP-9 (b) in tears from patients with dry eye and normal controls. Values were presented as mean ± SD. ^*∗∗*^Significant difference with *P* < 0.001.

The concentrations of MMP-2 and MMP-9 in the tears of all subjects are shown in Figure 2. The two MMPs tested were detected in all of the samples. The tear concentrations of MMP-2 and MMP-9 in the dry eye group were significantly greater than those in the control group (*P* < 0.001).

### 3.5. Correlation of Sex Steroid Levels with MMP-2 and MMP-9 Tear Concentrations and Clinical Tests

**Figure 3:**
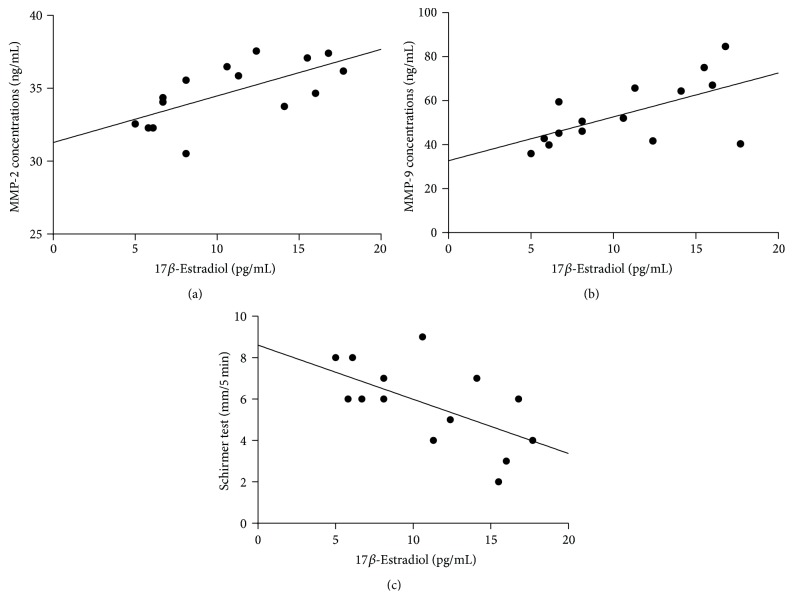
Correlation of 17*β*-estradiol levels with (a) tear MMP-2 concentrations (*ρ* = 0.67, *P* = 0.006); (b) tear MMP-9 concentrations (*ρ* = 0.58, *P* = 0.03); and (c) Schirmer test results (*ρ* = −0.58, *P* = 0.02). Spearman's rank correlation coefficients were calculated in 15 dry eye subjects who had detectable levels of 17*β*-estradiol.

Table 3 shows the correlation of sex steroids levels with MMP-2 and MMP-9 tear concentrations and clinical tests in the dry eye group. Specifically, the results of the correlation between 17*β*-estradiol and other test parameters were calculated from the data gathered from 15 dry eye subjects who had detectable levels of 17*β*-estradiol. The analysis of the other three sex steroids was conducted in all 44 dry eye subjects. The levels of 17*β*-estradiol positively correlated with tear concentrations of MMP-2 and MMP-9. The results of Schirmer test showed a significant negative correlation with 17*β*-estradiol (Figure 3). The levels of testosterone showed a weak negative correlation with TBUT results but no correlation with the results of other tests. The levels of DHEA-S and progesterone showed no correlations with the results of the other tests.

## 4. Discussion

The prevalence of dry eye is higher in females, especially in postmenopausal women [1]. This fact indicates that sex steroid imbalance is related to the onset and development of dry eye. However, the role of sex steroids in dry eye is complex and remains to be fully understood. Our results demonstrate that the serum levels of 17*β*-estradiol were significantly higher in postmenopausal women with dry eye than in controls, whereas the levels of testosterone, DHEA-S, and progesterone between the two groups were not significantly different.

Only a few studies have investigated the levels of sex steroids in postmenopausal women with dry eye. Tamer et al. [29] evaluated androgen levels in dry eye patients both with meibomian gland dysfunction (MGD) and without MGD and compared these levels with those of normal control subjects. Total testosterone levels were not significantly different among the three groups, which is consistent with our results. The study also reported lower levels of bioavailable testosterone, DHEA, and DHEA-S in MGD patients than in controls, whereas there was no significant difference between non-MGD dry eye patients and controls. Another small-sample study [30] also found no significant difference in total testosterone levels between postmenopausal women with dry eye and controls. Inconsistent with our findings, a recent study [31] reported that the serum levels of 17*β*-estradiol and total testosterone were significantly lower in evaporative dry eye patients than in controls. In patients with SS, the disease is not associated with significant alterations in serum levels of testosterone, estrone, or estradiol, whereas DHEA and DHEA-S levels were significantly reduced [32]. However, another study showed no significant difference in DHEA and DHEA-S levels in patients with SS [32, 33].

In our study, we did not subdivide dry eye patients into MGD or non-MGD categories. This could partially explain the differences in sex steroids levels between the current study and previous studies. It would be worth looking at aqueous deficient and evaporative dry eye separately. Other possible factors for the conflicting results among these studies are limited number of subjects, racial differences, and variations in the duration of menopause. In addition, serum sex steroids could not reflect the total estrogen and androgen pool in postmenopausal women [34]. It should be noted that humans are unique in possessing adrenal glands that secrete large amounts of DHEA and DHEA-S, which are then converted into androgens and estrogens by steroidogenic enzymes in peripheral tissues and thereby permit target tissues to adjust the amount of active sex hormones according to local requirements [34]. The human ocular surface has been shown to contain mRNAs for steroidogenic enzymes, which are necessary for the local synthesis and metabolism of androgens and estrogens [11]. Therefore, the human ocular surface may be among the many peripheral tissues and be a source of sex steroids.

Versura et al. [6] reported that ocular surface function impairment is greatest when estrogen levels are highest as this impairment occurs during the follicular phase in the menstrual cycle. We found that the levels of estrogen in dry eye patients were still higher than those of age-matched controls after menopause. In another study, 11 out of 20 asymptomatic postmenopausal women developed dry eye symptoms after three months of HRT (estrogen/progesterone) use, whereas symptomatic women were not relieved of dry eye by HRT [35]. A large population-based study of 25,665 postmenopausal women found an increased risk of dry eye in women using HRT, particularly among those using estrogen alone [8]. These data support the hypothesis that estrogen has detrimental effects on the ocular surface.

Inflammation is a common factor that underlies many causes of dry eye. This study assessed the ocular surface expression of MMP-2 and MMP-9, molecules strictly related to the inflammatory process [21]. MMP-9 activity has also been considered to be a better biomarker of dry eye disease severity than traditional clinical signs and is associated with disruption of corneal epithelial barrier function [36, 37].

In this study, quantitative real-time PCR showed that the conjunctival expression of MMP-9 was significantly higher in dry eye patients than in controls, similar to the results of Chotikavanich et al. [36], which found an increasing trend in MMP-9 expression in dry eye subjects stratified by severity level. However, we were unable to detect MMP-2 expression in the conjunctival epithelium in our present study, which is possibly explained by the lower MMP-2 production in the conjunctiva than in other ocular surface tissues [38]. In addition, a lower amount of total RNA was obtained by impression cytology, which may have decreased sensitivity.

Increased MMP-2 and MMP-9 production has been observed in the tear fluid collected from patients with systemic dry eye and from those with nonsystemic dry eye [37, 39]. Consistent with previous findings, we also found that the tear concentrations of MMP-2 and MMP-9 in dry eye subjects were significantly higher than those in controls.

We also observed that the levels of 17*β*-estradiol were correlated positively with tear concentrations of MMP-2 and MMP-9. This finding indicates that 17*β*-estradiol stimulates the activity of MMP-2 and MMP-9 in tears of patients with dry eye. The source of tear-derived MMP-2 and MMP-9 has not been established. Corneal epithelial cells [19] and lacrimal glands [21] have been found to synthesize both MMP-2 and MMP-9 in the ocular surface system. A recent study has shown that the major sources of tear-derived MMP-2 and MMP-9 following corneal wounding are the lacrimal gland and conjunctival-associated lymphoid tissue, whereas the corneal epithelium, stromal keratocytes, and conjunctival epithelium including goblet cells contribute little to tear-derived MMP-2 and MMP-9, and the meibomian glands do not appear to contribute at all [38]. Therefore, we postulate that 17*β*-estradiol may have effects on the promotion of inflammation in the lacrimal gland and conjunctival epithelium and may increase the activity of MMP-2 and MMP-9 in tears of patients with dry eye.

The rationale for our postulation is supported by previous studies on 17*β*-estradiol effects in ocular surface tissues. Suzuki and Sullivan have reported that 17*β*-estradiol upregulated the gene expression of proinflammatory cytokines and MMP-2, MMP-7, and MMP-9 in SV40 immortalized human corneal epithelial cells (HCEs) after 6 and/or 24 hours of hormone treatment [20]. However, 17*β*-estradiol effects on gene expression are not translated into changes in MMP-2 and MMP-9 activity in the culture of either SV40 HCEs or primary corneal epithelial cell cultures [40]. On the other hand, an animal study showed that estrogen treatment of ovariectomized rabbits significantly upregulates the expression and activity of MMP-2 and MMP-9 in the lacrimal gland [21]. A more recent study also demonstrated that systemic estradiol administration increases MMP-2 expression in lacrimal glands of ovariectomized rats [22]. Moreover, in the present study, 17*β*-estradiol was found to have a negative correlation with Schirmer test results but no correlation with the results of OSDI or corneal staining. Consistent with our result, Mathers et al. also reported a negative correlation between serum estradiol levels and tear production in postmenopausal women [4]. These results indicate that 17*β*-estradiol leads to regressive, inflammatory changes of the lacrimal gland, and, thus, tear production is reduced.

A limitation of our study is the fact that the method used to assess serum 17*β*-estradiol levels was not sensitive enough because many values were below the limit of detection. Further studies with larger subject numbers and with more sensitive analytical techniques are needed.

## 5. Conclusions

In conclusion, this study demonstrated that serum levels of 17*β*-estradiol were higher in postmenopausal women with dry eye than in controls. Levels of 17*β*-estradiol positively correlated with tear MMP-2 and MMP-9 concentrations and negatively correlated with Schirmer test results. We postulate that 17*β*-estradiol upregulates MMP-2 and MMP-9 production in the lacrimal gland and MMP-9 production in the conjunctival epithelium and thus increases the activity of MMP-2 and MMP-9 in tears of dry eye subjects. Our results support the findings from animal studies that showed upregulation of MMP levels following estradiol treatment.

## Figures and Tables

**Table 1:** Demographics and clinical characteristics in patients with dry eye and normal controls.

Characteristics	Dry eye group (*n* = 44)	Control group (*n* = 22)	*P* value
Age (years)	63.2 ± 7.4	60.7 ± 5.3	0.18
Duration of menopause (years)	11.2 ± 8.0	10.3 ± 5.9	0.81
BMI	22.9 ± 2.2	22.6 ± 2.1	0.70
Lipid profile			
Total cholesterol (mmol/L)	4.5 ± 0.8	4.7 ± 1.0	0.47
Triglycerides (mmol/L)	1.5 ± 0.6	1.6 ± 0.5	0.33
HDL-cholesterol (mmol/L)	1.1 ± 0.3	1.1 ± 0.2	0.36
LDL-cholesterol (mmol/L)	2.8 ± 0.8	3.0 ± 0.9	0.28
OSDI (points)	39.5 ± 24.4	14.0 ± 8.4	<0.001
Fluorescein TBUT (s)	2.5 ± 1.1	9.8 ± 3.9	<0.001
Corneal fluorescein staining (points)	3.1 ± 2.5	0.3 ± 0.4	<0.001
Schirmer test (mm/5 min)	7.1 ± 5.6	12.6 ± 6.2	<0.001

BMI = body mass index, HDL = high-density lipoprotein, LDL = low-density lipoprotein, OSDI = Ocular Surface Disease Index, and TBUT = tear film break-up time.

**Table 2:** Comparison of serum levels of sex steroids between the dry eye group and the normal control group.

Laboratory test	Dry eye group (*n* = 44)	Control group (*n* = 22)	*P* value
17*β*-Estradiol (pg/mL)			0.03^*∗*^
<5	29	20	
≥5	15	2	
Testosterone (nmol/L)	0.60 ± 0.41	0.40 ± 0.24	0.08
DHEA-S (*μ*mol/L)	3.53 ± 1.93	3.15 ± 1.30	0.57
Progesterone (nmol/L)	0.79 ± 0.53	0.63 ± 0.39	0.34

^*∗*^Pearson's chi-squared test.

**Table 3:** Spearman correlation of sex steroid levels with tear MMP concentrations and clinical tests.

Parameters	MMP-2		MMP-9		OSDI		TBUT		Corneal staining		Schirmer test	
	*ρ*	*P*	*ρ*	*P*	*ρ*	*P*	*ρ*	*P*	*ρ*	*P*	*ρ*	*P*
17*β*-Estradiol	0.67	0.006^*∗*^	0.58	0.03^*∗*^	−0.07	0.80	−0.38	0.17	−0.09	0.74	−0.58	0.02^*∗*^
Testosterone	0.28	0.12	0.21	0.24	0.14	0.41	−0.32	0.04^*∗*^	0.17	0.28	0.02	0.91
DHEA-S	0.004	0.98	0.16	0.38	0.31	0.06	−0.09	0.58	−0.16	0.32	−0.05	0.75
Progesterone	0.07	0.72	0.06	0.77	0.24	0.16	−0.15	0.34	−0.10	0.56	0.02	0.90

DHEA-S = dehydroepiandrosterone sulfate, MMP = matrix metalloproteinase, OSDI = Ocular Surface Disease Index, and TBUT = tear film break-up time.

^*∗*^Significant difference with *P* < 0.05

*ρ*: Spearman's correlation coefficient.

## References

[1] Smith J. A., Albenz J., Begley C. (2007). The epidemiology of dry eye disease: report of the Epidemiology Subcommittee of the International Dry Eye WorkShop (2007). *Ocular Surface*.

[2] McCarty C. A., Bansal A. K., Livingston P. M., Stanislavsky Y. L., Taylor H. R. (1998). The epidemiology of dry eye in Melbourne, Australia. *Ophthalmology*.

[3] Chia E.-M., Mitchell P., Rochtchina E., Lee A. J., Maroun R., Wang J. J. (2003). Prevalence and associations of dry eye syndrome in an older population: the Blue Mountains Eye study. *Clinical and Experimental Ophthalmology*.

[4] Mathers W. D., Stovall D., Lane J. A., Zimmerman M. B., Johnson S. (1998). Menopause and tear function: the influence of prolactin and sex hormones on human tear production. *Cornea*.

[5] Versura P., Campos E. C. (2005). Menopause and dry eye. A possible relationship. *Gynecological Endocrinology*.

[6] Versura P., Fresina M., Campos E. C. (2007). Ocular surface changes over the menstrual cycle in women with and without dry eye. *Gynecological Endocrinology*.

[7] Kramer P., Lubkin V., Potter W., Jacobs M., Labay G., Silverman P. (1990). Cyclic changes in conjunctival smears from menstruating females. *Ophthalmology*.

[8] Schaumberg D. A., Buring J. E., Sullivan D. A., Reza Dana M. (2001). Hormone replacement therapy and dry eye syndrome. *The Journal of the American Medical Association*.

[9] Rocha E. M., Wickham L. A., Da Silveira L. A. (2000). Identification of androgen receptor protein and 5*α*-reductase mRNA in human ocular tissues. *British Journal of Ophthalmology*.

[10] Esmaeli B., Harvey J. T., Hewlett B. (2000). Immunohistochemical evidence for estrogen receptors in meibomian glands. *Ophthalmology*.

[11] Schirra F., Suzuki T., Dickinson D. P., Townsend D. J., Gipson I. K., Sullivan D. A. (2006). Identification of steroidogenic enzyme mRNAs in the human lacrimal gland, meibomian gland, cornea, and conjunctiva. *Cornea*.

[12] Wickham L. A., Gao J., Toda I., Rocha E. M., Ono M., Sullivan D. A. (2000). Identification of androgen, estrogen and progesterone receptor mRNAs in the eye. *Acta Ophthalmologica Scandinavica*.

[13] Sullivan D. A., Sullivan B. D., Ullman M. D. (2000). Androgen influence on the meibomian gland. *Investigative Ophthalmology & Visual Science*.

[14] Sullivan D. A., Block L., Pena J. D. O. (1996). Influence of androgens and pituitary hormones on the structural profile and secretory activity of the lacrimal gland. *Acta Ophthalmologica Scandinavica*.

[15] Truong S., Cole N., Stapleton F., Golebiowski B. (2014). Sex hormones and the dry eye. *Clinical and Experimental Optometry*.

[16] Versura P., Giannaccare G., Campos E. C. (2015). Sex-steroid imbalance in females and dry eye. *Current Eye Research*.

[17] Na K.-S., Jee D. H., Han K., Park Y.-G., Kim M. S., Kim E. C. (2014). The ocular benefits of estrogen replacement therapy: a population-based study in postmenopausal Korean women. *PLoS ONE*.

[18] Jensen A. A., Higginbotham E. J., Guzinski G. M., Davis I. L., Ellish N. J. (2000). A survey of ocular complaints in postmenopausal women. *Journal of the Association for Academic Minority Physicians*.

[19] Li D.-Q., Lokeshwar B. L., Solomon A., Monroy D., Ji Z., Pflugfelder S. C. (2001). Regulation of MMP-9 production by human corneal epithelial cells. *Experimental Eye Research*.

[20] Suzuki T., Sullivan D. A. (2005). Estrogen stimulation of proinflammatory cytokine and matrix metalloproteinase gene expression in human corneal epithelial cells. *Cornea*.

[21] Zylberberg C., Seamon V., Ponomareva O., Vellala K., Deighan M., Azzarolo A. M. (2007). Estrogen up-regulation of metalloproteinase-2 and -9 expression in rabbit lacrimal glands. *Experimental Eye Research*.

[22] Song X., Zhao P., Wang G., Zhao X. (2014). The effects of estrogen and androgen on tear secretion and matrix metalloproteinase-2 expression in lacrimal glands of ovariectomized rats. *Investigative Ophthalmology and Visual Science*.

[23] Wingrove C. S., Garr E., Godsland I. F., Stevenson J. C. (1998). 17*β*-Oestradiol enhances release of matrix metalloproteinase-2 from human vascular smooth muscle cells. *Biochimica et Biophysica Acta (BBA)—Molecular Basis of Disease*.

[24] Potier M., Elliot S. J., Tack I. (2001). Expression and regulation of estrogen receptors in mesangial cells: influence on matrix metalloproteinase-9. *Journal of the American Society of Nephrology*.

[25] Schiffman R. M., Christianson M. D., Jacobsen G., Hirsch J. D., Reis B. L. (2000). Reliability and validity of the ocular surface disease index. *Archives of Ophthalmology*.

[26] Lemp M. A. (1995). Report of the National Eye Institute/Industry workshop on clinical trials in dry eyes. *The CLAO Journal*.

[27] Miller W. L., Auchus R. J. (2011). The molecular biology, biochemistry, and physiology of human steroidogenesis and its disorders. *Endocrine Reviews*.

[28] Singh R., Joseph A., Umapathy T., Tint N. L., Dua H. S. (2005). Impression cytology of the ocular surface. *British Journal of Ophthalmology*.

[29] Tamer C., Oksuz H., Sogut S. (2006). Androgen status of the nonautoimmune dry eye subtypes. *Ophthalmic Research*.

[30] Duarte M. C. B., Pinto N. T., Moreira H., Moreira A. T. R., Wasilewski D. (2007). Total testosterone level in postmenopausal women with dry eye. *Arquivos Brasileiros De Oftalmologia*.

[31] Gagliano C., Caruso S., Napolitano G. (2014). Low levels of 17-*β*-oestradiol, oestrone and testosterone correlate with severe evaporative dysfunctional tear syndrome in postmenopausal women: a case-control study. *British Journal of Ophthalmology*.

[32] Sullivan D. A., Bélanger A., Cermak J. M. (2003). Are women with Sjögren's syndrome androgen-deficient?. *Journal of Rheumatology*.

[33] Brennan M. T., Sankar V., Leakan R. A. (2003). Sex steroid hormones in primary Sjögren's syndrome. *The Journal of Rheumatology*.

[34] Labrie F., Bélanger A., Luu-The V. (1998). DHEA and the intracrine formation of androgens and estrogens in peripheral target tissues: its role during aging. *Steroids*.

[35] Erdem U., Ozdegirmenci O., Sobaci E., Sobaci G., Göktolga U., Dagli S. (2007). Dry eye in post-menopausal women using hormone replacement therapy. *Maturitas*.

[36] Chotikavanich S., de Paiva C. S., de Quan Li (2009). Production and activity of matrix metalloproteinase-9 on the ocular surface increase in dysfunctional tear syndrome. *Investigative Ophthalmology & Visual Science*.

[37] Vandermeid K. R., Su S. P., Ward K. W., Zhang J.-Z. (2012). Correlation of tear inflammatory cytokines and matrix metalloproteinases with four dry eye diagnostic tests. *Investigative Ophthalmology and Visual Science*.

[38] Petznick A., Madigan M. C., Garrett Q., Sweeney D. F., Evans M. D. M. (2013). Contributions of ocular surface components to matrix-metalloproteinases (MMP)-2 and MMP-9 in feline tears following corneal epithelial wounding. *PLoS ONE*.

[39] Solomon A., Dursun D., Liu Z., Xie Y., Macri A., Pflugfelder S. C. (2001). Pro- and anti-inflammatory forms of interleukin-1 in the tear fluid and conjunctiva of patients with dry-eye disease. *Investigative Ophthalmology and Visual Science*.

[40] Suzuki T., Sullivan D. A. (2006). Comparative effects of estrogen on matrix metalloproteinases and cytokines in immortalized and primary human corneal epithelial cell cultures. *Cornea*.

